# Single Nucleotide Polymorphisms of CYP3A4 and CYP3A5 in Romanian Kidney Transplant Recipients: Effect on Tacrolimus Pharmacokinetics in a Single-Center Experience

**DOI:** 10.3390/jcm13071968

**Published:** 2024-03-28

**Authors:** Corina Andreea Rotarescu, Ion Maruntelu, Ion Rotarescu, Alexandra-Elena Constantinescu, Ileana Constantinescu

**Affiliations:** 1 Immunology and Transplant Immunology, Carol Davila University of Medicine and Pharmacy, 258 Fundeni Avenue, 022328 Bucharest, Romania; corina.rotarescu@drd.umfcd.ro (C.A.R.); alexandra-elena.constantinescu0720@stud.umfcd.ro (A.-E.C.); ileana.constantinescu@imunogenetica.ro (I.C.); 2 Centre of Immunogenetics and Virology, Fundeni Clinical Institute, 258 Fundeni Avenue, 022328 Bucharest, Romania; 3 Department of Cardiovascular Surgery, Prof. Dr. C. C. Iliescu Emergency Institute for Cardiovascular Diseases, 258 Fundeni Avenue, 022328 Bucharest, Romania; rtrsc_i@yahoo.com; 4 Academy of Romanian Scientists (AOSR), 3 Ilfov Street, 030167 Bucharest, Romania

**Keywords:** CYP3A4*1.001, single nucleotide polymorphisms (SNPs), tacrolimus (Tac), C0/D ratio

## Abstract

**Background**: This study examines the impact of CYP3A4 and CYP 3A5 genotypes on tacrolimus (Tac) pharmacokinetics in Romanian kidney transplanted patients. **Methods:** We included 112 kidney recipients genotyped for CYP3A5*3, CYP3A4*1.001, and CYP3A4*22. Patients were categorized into poor, intermediate, rapid, and ultra-rapid metabolizers based on the functional defects linked to CYP3A variants. **Results:** Predominantly male (63.4%) with an average age of 40.58 years, the cohort exhibited a high prevalence of the CYP3A4*1/*1 (86.6%) and CYP3A5*3/*3 (77.7%) genotypes. CYP3A4*1.001 and CYP3A5*1 alleles significantly influenced the Tac concentration-to-dose (C0/D) ratio in various post-transplant periods, while the CYP3A4*22 allele showed no such effect (*p* = 0.016, *p* < 0.001). Stepwise regression highlighted the CYP3A4*1.001’s impact in early post-transplant phases, with hematocrit and age also influencing Tac variability. **Conclusions**: The study indicates a complex interaction of CYP3A4 and CYP3A5 genotypes on Tac metabolism, suggesting the necessity for personalized medication approaches based on genetic profiling in kidney transplant recipients.

## 1. Introduction

Immunosuppression is the treatment of choice for kidney transplant recipients to avoid rejection and the loss of transplanted kidneys. The development of new immunosuppressive drugs has expanded therapeutic options, reduced adverse reactions, and simplified long-term care for kidney transplant recipients. It is important to note that transplant centers worldwide follow different protocols for administering immunosuppressive drugs.

Tacrolimus (Tac) is a macrolide lactone isolated from *Streptomyces tsukubaensis* [1]. It functions by inhibiting T-cell activation and the production of cytokines, ultimately helping in the prevention of graft rejection. Tac has a high affinity for erythrocytes and binds to albumin, proteins, and α-1-acid glycoproteins [2]. The distribution of Tac between plasma and whole blood is influenced by several variables, including the temperature at which plasma is separated, the concentration of the drug, and the concentration of plasma proteins [2].

Tac is extensively metabolized in the gastrointestinal tract and liver, utilizing the cytochrome P450 enzymes [3,4]. Therefore, the majority of the variability in Tac pharmacokinetics may be explained by allelic variation in the CYP3A5 and CYP3A4 genes. The CYP3A5*3 allele is a mutant allele with a mutation in intron 3, resulting from deficient mRNA splicing and limited conversion of a functional protein [5]. The CYP3A5*1 allele, being a functional allele, is associated with increased Tac metabolism. Consequently, individuals expressing this allele (homozygous *1/*1 or heterozygous *1/*3), referred to as “expressers”, require higher doses of Tac to achieve therapeutic drug levels compared to non-expressers (CYP3A5*3/*3 genotype) [6,7]. The CYP3A4*1.001 (previously known as CYP3A4*1B) allele [8] is associated with a specific genetic variation involving a T-to-C substitution in the promoter region of the CYP3A4 gene [9]. CYP3A4*22 is a mutant allele that results from a G-to-A allele substitution (rs35599367) [10]. It is associated with a decrease in the expression or enzyme activity of CYP3A4 and has a slow metabolism rate and an increased risk of adverse reactions [11].

Pharmacokinetic studies with Tac have shown significant inter- and intra-individual differences in its kinetics in kidney transplant patients [12]. It is critical to obtain and maintain a therapeutic level of immunosuppressants using adequate dosages, especially during the immediate post-transplant period. In clinical practice, to optimize the balance between graft rejection risk and immunosuppressive drug toxicity, the therapeutic drug monitoring strategy has been the measurement of Tac blood concentration (C0). According to the CPIC dosing guidelines, they recommend increasing the starting dose to 1.5 to 2 times that of the recommended starting dose, with a total starting dose of no more than 0.3 mg/kg/day, while subsequent doses should be determined based on the therapeutic drug monitoring strategy [13]. The Dutch Pharmacogenetics Working Group (DPWG) recommends a dose of 1.5 times normal for heterozygote expressers and 2.5 times normal for homozygous expressers. Also, subsequent dosing should be based on the therapeutic drug monitoring strategy [14]. The International Association of Therapeutic Drug Monitoring and Clinical Toxicology (IATDMCT) presents information on genotype-based Tac doses [15]. While genotype-based dosing has shown promise in tailoring initial Tac doses based on an individual’s genetic profile, the clinical evidence supporting its significant impact on improving clinical outcomes after solid organ transplantation has been inconclusive. All these expert groups have highlighted that while pharmacogenetic testing, particularly involving genes like CYP3A5 and CYP3A4, can help predict an individual’s ability to metabolize Tac, there may be limitations regarding its direct impact on clinical outcomes post-transplantation. It is important to note that pharmacogenomics and its applications in personalized medicine are evolving. The conclusions and recommendations may change as more evidence from larger, well-designed studies becomes available.

We have selected to investigate CYP3A5*3, CYP3A4*22, and CYP3A4*1.001 since there is published evidence that these single nucleotide polymorphisms (SNPs) influence Tac dose requirements.

This observational and retrospective single-center study aimed to analyze the association between the CYP3A SNPs and Tac pharmacokinetics in the first year after kidney transplantation.

## 2. Materials and Methods

### 2.1. Patients

A retrospective analysis was conducted on 112 kidney transplant recipients treated between 2019 and 2022 at the Fundeni Clinical Institute, Bucharest. The exclusion criteria included retransplants, those aged under 18 years, a history of hepatitis B and C virus infections, immunization with anti-HLA class I and class II antibodies, and patients with a history of chronic use of substances known to interfere with Tac absorption, distribution, metabolism, and excretion (macrolides, rifampicin, phenytoin, and carbamazepine). All patients included in the study were treated with Tac in the first year after transplantation. A written informed consent was obtained from each of the patients. The study was approved by the Ethical Committee of Fundeni Clinical Institute (No. 61439/24 November 2023).

### 2.2. Immunosuppressive Therapy

All patients received induction therapy with an anti-interleukin-2 receptor monoclonal antibody (Basiliximab 20 mg on days 0 and 4) or with anti-thymocyte globulin (ATG 1.5 mg/kg/day during the first four days). The immunosuppressive maintenance therapy after renal transplantation consisted of triple therapy based on immediate-release Tac (*Prograf) or extended-release Tac (*Advagraf), mycophenolate mofetil, and prednisone. Based on the internal protocol in the Nephrology Clinical Ward from Fundeni Clinical Institute, all the patients had the initial Tac dose of 0.15–0.20 mg/kg, administered orally once daily or twice daily. Afterward, clinicians adjust doses to achieve target Tac blood levels: 12–15 ng/mL in the first 14 days, 10–12 ng/mL during 15–30 days, 8–10 ng/mL during 31–60 days, and 6–8 ng/mL after 60 days. These adjustments considered patient comorbidities, the risk of rejection, and calcineurin inhibitor nephrotoxicity.

### 2.3. Tac Blood Levels

Tac C0 levels were assessed before administering the morning doses at 2 weeks in the first month, then monthly until the end of the first year of post-kidney transplantation. The quantitative determination of Tac concentrations in human whole blood was performed using the chemiluminescent microparticle immunoassay (CMIA) technology with the Architech System^®^ i2000 (Abbott, Abbott Diagnostic, Green Oaks, IL, USA). The measuring range is 2 ng/mL to 30 ng/mL.

### 2.4. DNA Extraction and Genotyping

Following the manufacturer’s instructions, we used whole blood collected in EDTA-coated vacutainers for DNA extraction. Genomic DNA was extracted using the QIAmp DNA Blood Mini Kit (Qiagen, Hilden, Germany). A Nanophotometer Implen P300 spectrophotometer was used to measure the concentration and purity of DNA samples. 

The recipients were genotyped for CYP3A4*1.001 (rs2740574, T = CYP3A4*1, C = CYP3A4*1.001), CYP3A4*22 (rs35599367, G = CYP3A4*1, A = CYP3A4*22), and CYP3A5*3 (rs776746, T = CYP3A5*1, C = CYP3A5*3) alleles using TaqMan^®^ Drug Metabolism Genotyping Assays (Life Technologies Corporation, Pleasanton, CA, USA). SNP genotyping was performed on the Applied Biosystems 7300 Real-Time PCR System (7300 System v.1.4.0). The final volume for 96 reactions was 25 µL, consisting of 12.5 µL TaqMan Universal PCR Master Mix, 1.25 µL TaqMan Genotyping Assay Mix, and 11.25 µL DNA. The PCR program consisted of an initial denaturation step of 10 min at 95 °C and 50 cycles at 92 °C for 15 s and 60 °C for 1 min. After PCR amplification, we performed an endpoint plate read on Applied Biosystems 7300 using the Sequence Detection System Software Core Application.

### 2.5. Statistical Analysis

We used IBM SPSS version 20 for conducting statistical analyses. For variables demonstrating a normal distribution, we reported mean ± standard deviation (SD), while for those exhibiting an abnormal distribution, we utilized median and interquartile range (IQR). Qualitative variables’ frequency and percentage were examined using Fisher’s exact or Chi-Square tests. Categorical data was expressed as percentages. To compare two groups, the Mann-Whitney U test was employed, while the Kruskal-Wallis test assessed more than two groups. We used the formula described by Kang et al. [16] to compute D′ values for linkage disequilibrium (LD).

The C0/D ratio was calculated using the formula: C0/D ratio = Tac C0 (ng/mL)/Tac dose (mg/kg). All available Tac blood levels for a patient were included in the statistical analysis. To explore the impact of multiple factors on the C0/D ratio variability, we used stepwise linear regression. The analysis considered only the rare variants *1.001 and *22 of CYP3A4 and CYP3A5*1 and additional parameters such as age, albumin, and hematocrit. The mean Tac C0 levels were compared with the mentioned range values for each studied period. The time therapeutic range (TTR) was calculated using the Rosendaal method [17]. The Wilcoxon test was utilized to compare TTR between two groups (with and without complications). Additionally, variations in survival curves were assessed using the log-rank test. A multivariate Cox proportional hazard model was used to evaluate the influence of Tac C0 levels and TTR on complications. Adjusted factors included recipient ages and the CYP3A gene profile. Statistical significance was defined as *p*-values < 0.05.

## 3. Results

### 3.1. Demographic Characteristics of Patients

The recipients had an average age of 40.58 ± 11.05 years and a mean body mass index (BMI) of 23.8 ± 4.09, with the majority being male (*n* = 71). Most patients (89.3%) were on hemodialysis, and 29.5% presented with hypertension on admission, while 13.4% had diabetes. The major cause of transplantation was IgA nephropathy (16.1%). Table 1 illustrates other causes of chronic kidney disease. Regarding transplant characteristics, the median cold ischemia time was 10.9 h. Most patients (98.2%) underwent induction therapy with Basiliximab, and 66.1% required maintenance therapy with Prograf.

The predominant genotype among the patients was CYP3A4*1/*1, with the absence of the CYP3A4*1.001/*1.001 and CYP3A4*22/*22 genotypes. Notably, 78.6% of individuals carried the CYP3A4*1/*22 genotype. In the case of CYP3A5, the vast majority were homozygous for the CYP3A5*3/*3 variant. 

According to the observed variability of pharmacokinetics, we separated the patients into four groups, using the same approach as Hannachi et al.’s research: (1) poor metabolizers: CYP3A4*22 carriers with CYP3A5*3/*3; (2) intermediate metabolizers: CYP3A4*22 carriers plus one CYP3A5*1 allele or CYP3A4*1/*1 plus CYP3A5*3/*3; (3) rapid metabolizers: CYP3A4*1.001 allele in combination with CYP3A5*3/*3 or CYP3A4*1/*1 carriers with CYP3A5*1 allele; and (4) ultra-rapid metabolizers: CYP3A4*1.001 allele in combination with CYP3A5*1 carriers or CYP3A4*1.001/*1.001 [18]. Five patients did not meet the criteria for any of the metabolization groups defined by Hannachi et al. and therefore were not included in these groups. The frequencies of the studied genotypes and metabolizer status groups are shown in Table 2.

An analysis of the variants in our study revealed that there were strong linkages between pairs of alleles from different loci: CYP3A4*1.001 and CYP3A5*3 alleles (D′ = 1), CYP3A4*22 and CYP3A5*3 allele pairs (D′ = 0.95), or between CYP3A4*1.001 and CYP3A5*1 (D′ = 0.77). On the other hand, a weak linkage was between the CYP3A4*22 and CYP3A5*1 allele pairs (D′ = 0.01). Additionally, there was a negative D′ value in the case of CYP3A4*1.001 and CYP3A4*22 allele pairs (D′ = −0.32).

### 3.2. CYP3A Genotype and C0/D Ratio over Time Intervals

The mean Tac doses received during specific post-transplant periods (1–14 days, 15–30 days, 31–60 days, and beyond 60 days) were 13.02 ± 3.34, 12.62 ± 4.69, 9.58 ± 4.55, and 4.78 ± 1.99 mg/kg, respectively. The overall average dose was 9.45 ± 1.04 mg/kg. Corresponding C0 averages for these periods were 12.21 ± 3.30, 14.10 ± 3.38, 12.32 ± 3.21, and 7.79 ± 0.72 ng/mL, with a global average of 7.23 ± 2.46 ng/mL. The C0/D ratio for these periods was 69.28 ± 31.96, 88.06 ± 46.44, 108.25 ± 63.02, and 134.56 ± 74.79 ng/mL per mg/kg, respectively, with a global average ratio of 52.89 ± 21.88 ng/mL per mg/kg.

Regardless of the genotype group, the C0/D ratio was observed to vary significantly at different post-transplant time intervals. However, the specific changes depended on the genotype and the phase of the post-transplant period.

**Figure 1:**
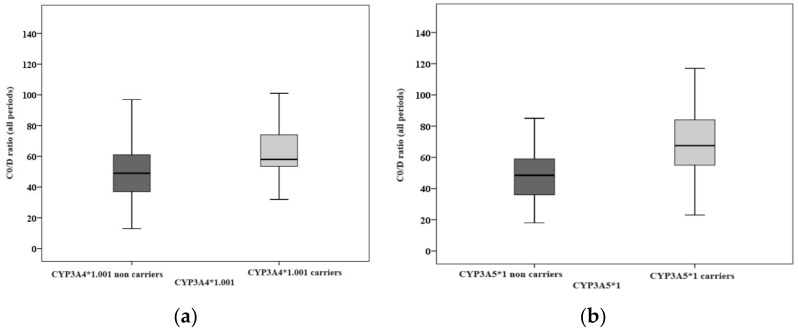
Analyzing Tac exposure between carriers and non-carriers of CYP3A4*1.001 (**a**) and CYP3A5*1 (**b**).

The statistical analyses were conducted using the Mann-Whitney U test. Concerning the CYP3A4*1.001 allele, the C0/D ratio exhibited a statistically significant difference between carriers and non-carriers across different periods (overall *p* = 0.016, during 1–14 days *p* = 0.015, during 31–60 days *p* = 0.011, and beyond 60 days *p* = 0.015). In the case of the CYP3A5*1 allele, a significant difference in the C0/D ratio was observed between carriers (CYP3A5*1/*1 and CYP3A5*1/*3) and individuals with the CYP3A5*3/*3 genotype during all post-transplant phases (*p* < 0.001) (Figure 1). For the CYP3A4*22 allele, the C0/D ratio did not differ significantly between non-carriers and carriers (*p* = 0.413). 

When analyzing the demographic and clinical parameter variation based on the metabolizer status, no statistically significant differences were found in age and weight across all metabolizer clusters. 

### 3.3. Effects of Various Clinical and Laboratory Parameters on C0/D Ratio over Time Intervals

A stepwise regression model was employed to assess the impact of various clinical and laboratory parameters on the variability of the C0/D ratio, both regardless of and according to the post-transplant period.

Table 3 illustrates the influence of these independent parameters on the normalized dose of Tac during different post-transplant periods.

In the early post-transplant phase (1–14 days), the CYP3A4*1.001 polymorphism showed a significant correlation with the Tac C0/D ratio, explaining 21.3% of its variability (*p* = 0.009). At the same time, CYP3A4*22 and CYP3A5*1 did not demonstrate a significant association. Hematocrit is also strongly correlated with the C0/D ratio in this phase, accounting for 8.8% of the variability (*p* = 0.002).

During the 15–30-day post-transplant period, hematocrit maintained a strong and significant correlation (partial r^2^ = 0.130, *p* < 0.001). Similarly, age was significantly correlated, explaining 18.4% of the Tac variability. 

In the 31–60-day post-transplant period, the CYP3A4*1.001 allele maintained a significant correlation with the Tac C0/D ratio (partial r^2^ = 0.140, *p* = 0.010), and hematocrit again demonstrated a strong association, accounting for 8% of the variability (*p* = 0.004). Additionally, age (partial r^2^ = 0.177, *p* = 0.036) showed a significant association with Tac variability. 

Beyond 60 days post-transplant, the CYP3A4*1.001 allele significantly correlated with the Tac C0/D ratio (partial r^2^ = 0.230, *p* = 0.022). The results indicated that hematocrit and age continued to be highly associated, accounting for 11.9% and 19.2% of the variability (*p* < 0.001 and *p* = 0.002).

The regression model showed that CYP3A4*1.001, hematocrit, and age were identified as significant variables over the entire period.

### 3.4. Therapeutic Range of Tac Level

Patients receiving Tac were classified based on whether their C0 levels fell within or outside the therapeutic range at different post-transplant periods. Specifically, the therapeutic range was set at 12–15 ng/mL during the 1–14-day post-transplant period, 10–12 ng/mL during the 15–30 days, 8–10 ng/mL during the 31–60 days, and 6–8 ng/mL after 60 days (Table 4). The influence of different genotypes on this categorization was examined across various genetic groups.

For the CYP3A4*1.001 genotype, during the first 14 days post-transplant (therapeutic range: 12–15 ng/mL), there was no significant difference in the proportion of patients falling inside or outside the therapeutic range (*p* = 0.492). This lack of significant influence of the CYP3A4*1.001 genotype on Tac levels persisted in subsequent periods: 15–30 days (*p* = 0.533), 31–60 days (*p* = 0.292), and beyond 60 days (*p* = 0.916).

In contrast, for the CYP3A4*22 genotype, a significant difference emerged during the 31–60-day post-transplant period (therapeutic range: 8–10 ng/mL), with a *p*-value of 0.035, indicating that this genotype might influence Tac levels specifically during this time frame. However, for the combined CYP3A4*1.001/*22 genotype, no significant differences were observed in any of the post-transplant periods across the varying therapeutic ranges (*p*-values: 0.854 for 1–14 days, 0.220 for 15–30 days, 0.185 for 31–60 days, and 0.436 for >60 days).

Regarding the CYP3A5 genotype, no significant differences were observed in the proportion of patients within or outside the therapeutic range in any of the periods (*p*-values: 0.486 for 1–14 days, 0.190 for 15–30 days, 0.420 for 31–60 days, and 0.103 for >60 days).

In summary, while certain genotypes, like CYP3A4*22, showed a significant association with Tac levels during the 31–60-day post-transplant period, other genotypes did not consistently influence the different therapeutic ranges defined for each post-transplant phase.

**Figure 2:**
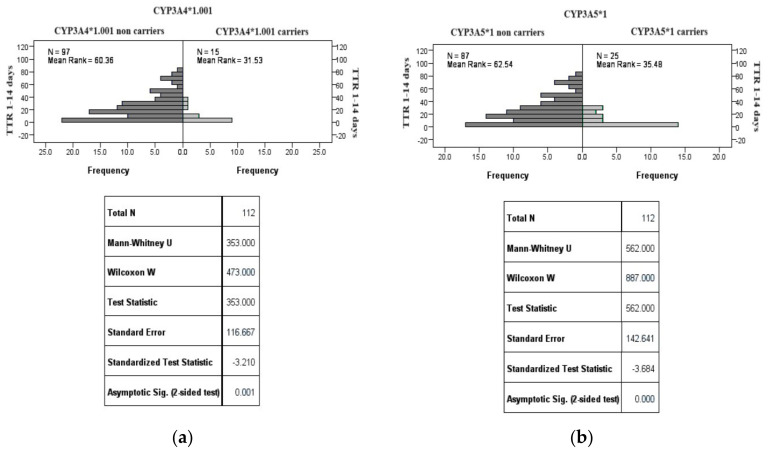
TTR at 1−14 days across carriers and non-carriers of CYP3A4*1.001 (**a**) and CYP3A5*1 (**b**).

TTR levels were significantly lower in patients carrying the CYP3A4*1.001 (*p* = 0.001) and CYP3A5*1 genes (*p* < 0.001) within the 1–14-day period compared to those who do not carry these genes (Figure 2).

There was no significant interaction effect between genotype status and therapeutic range on TTR. This means that the effect of being a genotype carrier on TTR is not different for subjects within or beyond the 60-day post-event range.

Lastly, when considering the broader categorization of metabolizer status, no significant association was found between metabolizer groups and being within or outside the therapeutic range of Tac levels in any of the post-transplant periods (*p*-values: 0.783 for 1–14 days, 0.283 for 15–30 days, 0.040 for 31–60 days, and 0.341 for >60 days).

## 4. Discussion

Kidney transplant recipients treated with a combination of drugs respond to immunosuppressive drugs in different ways. In this study, we investigated the influence of genetic variations, including CYP3A5*1, CYP3A5*3, CYP3A4*22, and CYP3A4*1.001, on Tac pharmacokinetics among Romanian kidney transplant recipients during their first year post-transplant. We aimed to analyze the association between these genetic polymorphisms and Tac dose requirements, with the ultimate goal of contributing to the advancement of personalized immunosuppressive therapy, thereby improving the efficacy and safety of kidney transplantation. 

Our findings revealed intricate connections between specific genetic variations in CYP3A4 and the pharmacokinetics of Tac across different post-transplantation periods. Additionally, we explored the impact of age and hematocrit on Tac exposure in Romanian kidney transplant patients.

The CYP3A4*1.001 allele is present in 13.4% of our patients, indicating that they are predominantly carriers of CYP3A4*1/*1. The majority of our patients are carriers of CYP3A5*3/*3 and CYP3A5*1/*3. Our data align with those of Caucasian and Asian populations [19,20,21,22,23]. 

Our study revealed strong LD between CYP3A4*1.001 and CYP3A5*1 [4,24], CYP3A4*1.001 and CYP3A5*3 [25,26,27], or CYP3A4*22 and CYP3A5*3. However, the effect of CYP3A4*1.001 is not influenced by CYP3A5, C0/D being significantly reduced in CYP35, independently of the CYP3A4 variant [18,28]. The negative D′ value for CYP3A4*1.001 and CYP3A4*22 allele pairs (D′ = −0.32) indicates that these variants are less likely to be inherited together.

Our findings indicate a statistically significant difference in the C0/D ratio between carriers and non-carriers of the CYP3A4*1.001 alleles, with *p*-values of 0.016. These genetic variations are associated with higher Tac exposure, which differs from other studies conducted in Tunisia [29]. 

The CYP3A5*1 allele also significantly impacted the C0/D ratio, particularly between carriers and individuals with the CYP3A5*3/*3 genotype (*p* < 0.001). These findings align with studies conducted by Thervet et al. and MacPhee et al. They discovered that individuals with the CYP3A5*1 genotype exhibited a higher hepatic and intestinal metabolism rate, necessitating a larger daily dosage to obtain sufficient Tac blood levels [30,31]. Aouam et al. observed that patients with CYP3A5*1 carriers required significantly higher Tac doses, specifically during the early post-transplant phase (1 to 90 days) [28]. 

In contrast to our findings, Wanas et al. reported that individuals with the CYP3A4*1/*22 genotype had significantly higher C0/D at day 10, and there was no significant difference between the CYP3A4*1/*1 and CYP3A4*1/*22 genotypes in terms of trough C0 levels at different time points [32].

The study also observed significant differences in the C0/D ratio when comparing metabolizer statuses. Our patients were predominantly poor metabolizers (69.6%), possibly because CYP3A4*1/*22 was present in 78.6% of our patients and only 16.1% were intermediate metabolizers. This result differs from the Spanish, Polish, and German populations, where it was observed that intermediate metabolizers were more common and slow metabolizers were less common [18,33,34,35]. 

The metabolizer status, particularly at the extremes of ultra-rapid and poor metabolizers, influences the C0/D ratio. At the same time, demographic factors such as age and weight did not exhibit significant variations across different metabolizer groups. This underscores the complexity of the relationship between metabolizer status and Tac metabolism. 

Through a stepwise regression model, we observed that certain genetic factors consistently influence the Tac C0/D ratio across various post-transplant periods. The CYP3A4*1.001 variant explains a considerable percentage of this variability (21.3% during the first weeks, 14% during the second month, and 23% after 60 days), indicating a significant difference in Tac C0/D between CYP3A4*1.001 carriers and non-carriers, especially during the early post-transplant period. These findings are inconsistent with recent results by Youssef et al. [24] and Hannachi et al. [18], who reported a significant difference in Tac trough per dose based on CYP3A5*3. It has been shown in previous studies [36,37,38] that the hematocrit of a patient can have an impact on the way drugs are metabolized. Therefore, in our improved model for predicting within-patient variability in Tac drug metabolism, we considered the clinical relevance of each factor, including CYP3A genotype and hematologic parameters.

In our study, hematocrit and age also play a crucial role, particularly in the later post-transplant phases. Similar to our study, Jonge et al. identified hematocrit as a predictor factor of the Tac C0/D ratio [39]. These findings underscore the importance of both genetic and clinical factors in managing Tac dosing in transplant patients. 

Furthermore, the study investigated the influence of different genotypes on patients Tac levels within defined therapeutic ranges during various post-transplant periods. Only the CYP3A4*22 genotype demonstrated a significant association with Tac levels during a specific post-transplant period (31–60 days). This partially differs from the findings of Hu et al. [40] and Santoro et al. [41], who reported a lack of influence of CYP3A4*22 on Tac exposure in the first months post-transplant. However, other genetic variations did not consistently impact Tac levels across the various therapeutic ranges.

According to our study, significant differences were observed only in TTR during the 1–14 days when comparing carriers and non-carriers of CYP3A4*1.001 and CYP3A5*1, as calculated using the Rosendaal method. Also, there was no association between TTR and metabolizer groups or any pot-transplant complications across any study period. The same results were also met in the study by Hernandez et al. on adult heart transplant recipients [42]. However, Liu et al. observed a generally reduced TTR (18–35%) throughout the lung transplant cohort [43]. The differences between our results and those of other similar studies could be related to the TTR calculation methodologies used [42,43]. 

Moreover, we studied the potential impacts of CYP3A4 and CYP3A5 genotypes on post-kidney transplant complications such as delayed graft function, rejection, BK infections, viral acute graft pyelonephritis, and suspected calcineurin inhibitor nephrotoxicity. Throughout the first year, we found no significant associations between the studied genes and the complications following kidney transplantation. Similarly, Sienkiewicz et al. in Polish patients could not find an association between CYP3A5 genotypes and calcineurin inhibitor drug levels at any investigated time point [44]. 

This study has some limitations. It is a retrospective analysis involving only Romanian patients, and there may be discrepancies in CYP3A genotype cluster activity compared to other studies. Our single-center study included a relatively small number of patients (*n* = 112), which may explain why we did not observe a significant impact of the tested genotypes on clinical outcomes.

## 5. Conclusions

The consistent significance of the CYP3A4*1.001 allele across different periods, along with hematocrit and age in the later stages, underscores the necessity for personalized Tac dosing strategies that consider these variables. Moreover, the variability in the influence of these factors over different post-transplant periods emphasized the dynamic nature of drug metabolism following transplantation, necessitating the importance of continuous monitoring and adjustment of drug dosing.

## Figures and Tables

**Table 1:** Demographic characteristics of patients.

Demographic Characteristics	Mean ± Standard Deviation
Age	40.58 ± 11.05
Male	71 (63.4%)
Female	41 (36.6%)
Body mass index (BMI) (kg/m^2^)	23.8 ± 4.09
**Comorbidities**	
Hypertension	33 (29.5%)
Diabetes	15 (13.4%)
Active/past smoker	26 (23.3%)
Hemodialysis	100 (89.3%)
Peritoneal dialysis	3 (2.7%)
**Chronic kidney disease causes**	
Undetermined etiology	45 (40.2%)
Glomerular disease	9 (8%)
Diabetes kidney disease	7 (6.3%)
Autosomal dominant polycystic kidney disease	7 (6.3%)
Tubulointerstitial disease	4 (3.6%)
IgA Nephropathy	18 (16.1%)
Nephritis due to vesicoureteral reflux	6 (5.4%)
Systemic lupus erythematosus	2 (1.8%)
Alport syndrome	6 (5.4%)
Wegener granulomatosis	1 (0.9%)
Hypertensive nephropathy	1 (0.9%)
Fabry disease	2 (1.8%)
ANCA positive Vasculitis	1 (0.9%)
Goodpasture syndrome	1 (0.9%)
Pyonephrosis	2 (1.8%)
**Laboratory parameters**	
Albumin (g/dL)	4.37 ± 0.48
Creatinine (mg/dL)	2.13 ± 0.94
eGFR (mL/min/1.73 m^2^)	48.31 ± 18.47
Urea (mg/dL)	72.2 ± 24.6
Hematocrit (%)	36.32 ± 4.26
**Transplant characteristics**	
Cold ischemia time (mean, mins)	656.94
Warm ischemia time (mean, mins)	37.12
HLA-MM (mean, range)	3 (0–6)
**Treatment characteristics**	
**Induction therapy**	
Anti-thymocite globuline	2 (1.8%)
Basiliximab	110 (98.2%)
**Maintenance therapy**	
Prograf	74 (66.1%)
Advagraf	38 (33.9%)
Tac concentration (C0) (ng/mL)	7.23 ± 2.46
Tac dose (mg/kg)	9.44 ± 1.04
Concentration/Dose (C0/D) Ratio (ng/mL per mg/kg)	52.89 ± 21.87
**Complications**	
Delayed graft function	9 (8%)
Rejection	4 (3.6%)
BK infections viral	7 (6.3%)
Acute graft pyelonephritis	3 (2.7%)
Suspected calcineurin inhibitor nephrotoxicity	1 (0.9%)

**Table 2:** Genotype frequency of patients.

Genotype	Total (*n* = 112)
CYP3A4*1.001 (rs2740574)	
TT (*1/*1)	97 (86.6%)
TC (*1/*1.001)	15 (13.4%)
CC (*1.001/*1.001)	0 (0%)
CYP3A5*3 (rs776746)	
TT (*1/*1)	7 (6.3%)
TC (*1/*3)	18 (16.1%)
CC (*3/*3)	87 (77.7%)
CYP3A4*22 (rs35599367)	
GG (*1/*1)	24 (21.4%)
GA (*1/*22)	88 (78.6%)
AA (*22/*22)	0 (0%)
Poor metabolizers	78 (69.6%)
Intermediate metabolizers	18 (16.1%)
Rapid metabolizers	1 (0.9%)
Ultra-rapid metabolizers	10 (8.9%)

**Table 3:** Effect of different parameters and allelic distribution on the normalized dose of Tac according to the post-transplant phase.

Post-Transplant Period (Days)	Independent Parameter	Partial r^2^	*p*-Value
1–14 days	CYP3A4*1.001	**0.213**	**0.009**
	CYP3A4*22	0.237	0.755
	CYP3A5*1	0.332	0.099
	**Age**	**0.236**	**0.008**
	**Hematocrit**	**0.088**	**0.002**
15–30 days	CYP3A4*1.001	0.204	0.116
	CYP3A4*22	0.207	0.774
	CYP3A5*1	0.277	0.173
	**Age**	**0.184**	**0.011**
	**Hematocrit**	**0.130**	**<0.001**
31–60 days	CYP3A4*1.001	**0.140**	**0.010**
	CYP3A4*22	0.181	0.504
	CYP3A5*1	0.220	0.406
	**Age**	**0.177**	**0.036**
	**Hematocrit**	**0.080**	**0.004**
>60 days	CYP3A4*1.001	**0.230**	**0.022**
	CYP3A4*22	0.232	0.809
	CYP3A5*1	0.287	0.272
	**Age**	**0.192**	**0.002**
	**Hematocrit**	**0.119**	**<0.001**
Regardless of post-transplant periods	CYP3A4*1.001	**0.247**	**0.023**
	CYP3A4*22	0.250	0.564
	CYP3A5*1	0.336	0.100
	**Age**	**0.210**	**0.004**
	**Hematocrit**	**0.149**	**<0.001**

*p*-value calculated with a stepwise regression model. Bold values are significant values.

**Table 4:** Patients in the therapeutic range based on genotype.

Post-Transplant Period (Days)	Genotype	Therapeutic Range (ng/mL)	*p*-Value
1–14	CYP3A4*1.001	12–15	0.492
15–30		10–12	0.533
31–60		8–10	0.292
>60		6–8	0.916
1–14	CYP3A4*22	12–15	0.689
15–30		10–12	0.126
31–60		8–10	**0.035**
>60		6–8	0.526
1–14	CYP3A4*1.001/*22	12–15	0.854
15–30		10–12	0.220
31–60		8–10	0.185
>60		6–8	0.436
1–14	CYP3A5*3	12–15	0.639
15–30		10–12	0.314
31–60		8–10	0.189
>60		6–8	0.834

*p*-value calculated by Fisher’s test. Bold values are significant values.

## Data Availability

The dataset is available on request from the authors.

## References

[1] Kino T., Hatanaka H., Hashimoto M., Nishiyama M., Goto T., Okuhara M., Kohsaka M., Aoki H., Imanaka H. (1987). FK-506, a Novel Immunosuppressant Isolated from a Streptomyces. I. Fermentation, Isolation, and Physico-Chemical and Biological Characteristics. J. Antibiot..

[2] Størset E., Holford N., Midtvedt K., Bremer S., Bergan S., Åsberg A. (2014). Importance of hematocrit for a tacrolimus target concentration strategy. Eur J Clin Pharmacol..

[3] Lecointre K., Furlan V., Taburet A.M. (2002). In Vitro Effects of Tacrolimus on Human Cytochrome P450. Fundam. Clin. Pharmacol..

[4] Hesselink D.A., Bouamar R., Elens L., van Schaik R.H.N., van Gelder T. (2014). The Role of Pharmacogenetics in the Disposition of and Response to Tacrolimus in Solid Organ Transplantation. Clin. Pharmacokinet..

[5] Zhai Q., van der Lee M., van Gelder T., Swen J.J. (2022). Why We Need to Take a Closer Look at Genetic Contributions to CYP3A Activity. Front. Pharmacol..

[6] Terrazzino S., Quaglia M., Stratta P., Canonico P.L., Genazzani A.A. (2012). The Effect of CYP3A5 6986A>G and ABCB1 3435C>T on Tacrolimus Dose-Adjusted Trough Levels and Acute Rejection Rates in Renal Transplant Patients: A Systematic Review and Meta-Analysis. Pharmacogenet. Genom..

[7] Hesselink D.A., van Schaik R.H.N., van der Heiden I.P., van der Werf M., Gregoor P.J.H.S., Lindemans J., Weimar W., van Gelder T. (2003). Genetic Polymorphisms of the CYP3A4, CYP3A5, and MDR-1 Genes and Pharmacokinetics of the Calcineurin Inhibitors Cyclosporine and Tacrolimus. Clin. Pharmacol. Ther..

[8] PharmVar. https://www.pharmvar.org/gene/CYP3A4.

[9] Sata F., Sapone A., Elizondo G., Stocker P., Miller V.P., Zheng W., Raunio H., Crespi C.L., Gonzalez F.J. (2000). CYP3A4 Allelic Variants with Amino Acid Substitutions in Exons 7 and 12: Evidence for an Allelic Variant with Altered Catalytic Activity. Clin. Pharmacol. Ther..

[10] Wang D., Sadee W. (2012). The Making of a CYP3A Biomarker Panel for Guiding Drug Therapy. J. Pers. Med..

[11] Elens L., Bouamar R., Hesselink D.A., Haufroid V., van der Heiden I.P., van Gelder T., van Schaik R.H.N. (2011). A New Functional CYP3A4 Intron 6 Polymorphism Significantly Affects Tacrolimus Pharmacokinetics in Kidney Transplant Recipients. Clin. Chem..

[12] Venkataramanan R., Jain A., Cadoff E., Warty V., Iwasaki K., Nagase K., Krajack A., Imventarza O., Todo S., Fung J.J. (1990). Pharmacokinetics of FK 506: Preclinical and Clinical Studies. Transplant. Proc..

[13] Birdwell K.A., Decker B., Barbarino J.M., Peterson J.F., Stein C.M., Sadee W., Wang D., Vinks A.A., He Y., Swen J.J. (2015). Clinical Pharmacogenetics Implementation Consortium (CPIC) Guidelines for CYP3A5 Genotype and Tacrolimus Dosing. Clin. Pharmacol. Ther..

[14] Swen J.J., Nijenhuis M., de Boer A., Grandia L., Maitland-van der Zee A.H., Mulder H., Rongen G.A.P.J.M., van Schaik R.H.N., Schalekamp T., Touw D.J. (2011). Pharmacogenetics: From Bench to Byte—An Update of Guidelines. Clin. Pharmacol. Ther..

[15] Brunet M., van Gelder T., Åsberg A., Haufroid V., Hesselink D.A., Langman L., Lemaitre F., Marquet P., Seger C., Shipkova M. (2019). Therapeutic Drug Monitoring of Tacrolimus-Personalized Therapy: Second Consensus Report. Ther. Drug Monit..

[16] Kang J.T.L., Rosenberg N.A. (2019). Mathematical Properties of Linkage Disequilibrium Statistics Defined by Normalization of the Coefficient D = PAB − PApB. Hum. Hered..

[17] Rosendaal F.R., Cannegieter S.C., van der Meer F.J., Briët E. (1993). A Method to Determine the Optimal Intensity of Oral Anticoagulant Therapy. Thromb. Haemost..

[18] Hannachi I., Chadli Z., Kerkeni E., Kolsi A., Hammouda M., Chaabane A., Ben Fredj N., Touitou Y., Boughattas N.A., Aouam K. (2021). Influence of CYP3A Polymorphisms on Tacrolimus Pharmacokinetics in Kidney Transplant Recipients. Pharmacogenomics J..

[19] Lamba J.K., Lin Y.S., Schuetz E.G., Thummel K.E. (2002). Genetic Contribution to Variable Human CYP3A-Mediated Metabolism. Adv. Drug Deliv. Rev..

[20] Drögemöller B., Plummer M., Korkie L., Agenbag G., Dunaiski A., Niehaus D., Koen L., Gebhardt S., Schneider N., Olckers A. (2013). Characterization of the Genetic Variation Present in CYP3A4 in Three South African Populations. Front. Genet..

[21] Cheung C.Y., Op den Buijsch R.A.M., Wong K.M., Chan H.W., Chau K.F., Li C.S., Leung K.T., Kwan T.H., de Vrie J.E., Wijnen P.A. (2006). Influence of Different Allelic Variants of the CYP3A and ABCB1 Genes on the Tacrolimus Pharmacokinetic Profile of Chinese Renal Transplant Recipients. Pharmacogenomics.

[22] Xie H.-G., Wood A.J.J., Kim R.B., Stein C.M., Wilkinson G.R. (2004). Genetic Variability in CYP3A5 and Its Possible Consequences. Pharmacogenomics.

[23] van Schaik R.H.N., van der Heiden I.P., van den Anker J.N., Lindemans J. (2002). CYP3A5 Variant Allele Frequencies in Dutch Caucasians. Clin. Chem..

[24] Yousef A.-M., Qosa H., Bulatova N., Abuhaliema A., Almadhoun H., Khayyat G., Olemat M. (2016). Effects of Genetic Polymorphism in CYP3A4 and CYP3A5 Genes on Tacrolimus Dose Among Kidney Transplant Recipients. Iran. J. Kidney Dis..

[25] Semiz S., Dujić T., Ostanek B., Prnjavorac B., Bego T., Malenica M., Mlinar B., Marc J., Causević A. (2011). Analysis of CYP3A4*1B and CYP3A5*3 Polymorphisms in Population of Bosnia and Herzegovina. Med. Glas..

[26] Bojanic K., Kuna L., Bilic Curcic I., Wagner J., Smolic R., Kralik K., Kizivat T., Ivanac G., Vcev A., Wu G.Y. (2020). Representation of CYP3A4, CYP3A5 and UGT1A4 Polymorphisms within Croatian Breast Cancer Patients’ Population. Int. J. Environ. Res. Public. Health.

[27] Gervasini G., García-Martín E., Ladero J.M., Pizarro R., Sastre J., Martínez C., García M., Diaz-Rubio M., Agúndez J.A. (2007). Genetic Variability in CYP3A4 and CYP3A5 in Primary Liver, Gastric and Colorectal Cancer Patients. BMC Cancer.

[28] Aouam K., Kolsi A., Kerkeni E., Ben Fredj N., Chaabane A., Monastiri K., Boughattas N. (2015). Influence of Combined CYP3A4 and CYP3A5 Single-Nucleotide Polymorphisms on Tacrolimus Exposure in Kidney Transplant Recipients: A Study According to the Post-Transplant Phase. Pharmacogenomics.

[29] Ben-Fredj N., Hannachi I., Chadli Z., Ben-Romdhane H., Boughattas N.A., Ben-Fadhel N., Aouam K. (2020). Dosing Algorithm for Tacrolimus in Tunisian Kidney Transplant Patients: Effect of CYP 3A4*1B and CYP3A4*22 Polymorphisms. Toxicol. Appl. Pharmacol..

[30] Thervet E., Anglicheau D., King B., Schlageter M.-H., Cassinat B., Beaune P., Legendre C., Daly A.K. (2003). Impact of Cytochrome P450 3A5 Genetic Polymorphism on Tacrolimus Doses and Concentration-to-Dose Ratio in Renal Transplant Recipients. Transplantation.

[31] Macphee I.A.M., Fredericks S., Tai T., Syrris P., Carter N.D., Johnston A., Goldberg L., Holt D.W. (2002). Tacrolimus Pharmacogenetics: Polymorphisms Associated with Expression of Cytochrome P4503A5 and P-Glycoprotein Correlate with Dose Requirement. Transplantation.

[32] Wanas H., Kamel M.H., William E.A., Fayad T., Abdelfattah M.E., Elbadawy H.M., Mikhael E.S. (2023). The Impact of CYP3A4 and CYP3A5 Genetic Variations on Tacrolimus Treatment of Living-Donor Egyptian Kidney Transplanted Patients. J. Clin. Lab. Anal..

[33] Kurzawski M., Dąbrowska J., Dziewanowski K., Domański L., Perużyńska M., Droździk M. (2014). CYP3A5 and CYP3A4, but Not ABCB1 Polymorphisms Affect Tacrolimus Dose-Adjusted Trough Concentrations in Kidney Transplant Recipients. Pharmacogenomics.

[34] Abdel-Kahaar E., Winter S., Tremmel R., Schaeffeler E., Olbricht C.J., Wieland E., Schwab M., Shipkova M., Jaeger S.U. (2019). The Impact of CYP3A4*22 on Tacrolimus Pharmacokinetics and Outcome in Clinical Practice at a Single Kidney Transplant Center. Front. Genet..

[35] Lloberas N., Elens L., Llaudó I., Padullés A., van Gelder T., Hesselink D.A., Colom H., Andreu F., Torras J., Bestard O. (2017). The Combination of CYP3A4*22 and CYP3A5*3 Single-Nucleotide Polymorphisms Determines Tacrolimus Dose Requirement after Kidney Transplantation. Pharmacogenet. Genom..

[36] Andrews L.M., Hesselink D.A., van Schaik R.H.N., van Gelder T., de Fijter J.W., Lloberas N., Elens L., Moes D.J.A.R., de Winter B.C.M. (2019). A Population Pharmacokinetic Model to Predict the Individual Starting Dose of Tacrolimus in Adult Renal Transplant Recipients. Br. J. Clin. Pharmacol..

[37] Stratta P., Quaglia M., Cena T., Antoniotti R., Fenoglio R., Menegotto A., Ferrante D., Genazzani A., Terrazzino S., Magnani C. (2012). The Interactions of Age, Sex, Body Mass Index, Genetics, and Steroid Weight-Based Doses on Tacrolimus Dosing Requirement after Adult Kidney Transplantation. Eur. J. Clin. Pharmacol..

[38] Gijsen V., Mital S., Van Schaik R.H., Soldin O.P., Soldin S.J., van der Heiden I.P., Nulman I., Koren G., de Wildt S.N. (2011). Age and CYP3A5 Genotype Affect Tacrolimus Dosing Requirements after Transplant in Pediatric Heart Recipients. J. Heart Lung Transplant..

[39] de Jonge H., de Loor H., Verbeke K., Vanrenterghem Y., Kuypers D.R.J. (2011). In Vivo CYP3A Activity Is Significantly Lower in Cyclosporine-Treated as Compared with Tacrolimus-Treated Renal Allograft Recipients. Clin. Pharmacol. Ther..

[40] Hu R., Barratt D.T., Coller J.K., Sallustio B.C., Somogyi A.A. (2018). CYP3A5*3 and ABCB1 61A>G Significantly Influence Dose-Adjusted Trough Blood Tacrolimus Concentrations in the First Three Months Post-Kidney Transplantation. Basic. Clin. Pharmacol. Toxicol..

[41] Santoro A.B., Struchiner C.J., Felipe C.R., Tedesco-Silva H., Medina-Pestana J.O., Suarez-Kurtz G. (2013). CYP3A5 Genotype, but Not CYP3A4*1b, CYP3A4*22, or Hematocrit, Predicts Tacrolimus Dose Requirements in Brazilian Renal Transplant Patients. Clin. Pharmacol. Ther..

[42] Liu M., Hernandez S., Aquilante C.L., Deininger K.M., Lindenfeld J., Schlendorf K.H., Van Driest S.L. (2024). Composite CYP3A (CYP3A4 and CYP3A5) Phenotypes and Influences on Tacrolimus Dose Adjusted Concentration in Adult Heart Transplant Recipients. Pharmacogenomics J..

[43] Liu M., Shaver C.M., Birdwell K.A., Heeney S.A., Shaffer C.M., Van Driest S.L. (2022). Composite CYP3A Phenotypes Influence Tacrolimus Dose-Adjusted Concentration in Lung Transplant Recipients. Pharmacogenet. Genomics.

[44] Sienkiewicz B., Hurkacz M., Kuriata-Kordek M., Augustyniak-Bartosik H., Wiela-Hojeńska A., Klinger M. (2016). The Impact of CYP3A5 on the Metabolism of Cyclosporine A and Tacrolimus in the Evaluation of Efficiency and Safety of Immunosuppressive Treatment in Patients after Kidney Transplantation. Pharmazie.

